# Accumulation of TOX high mobility group box family member 3 promotes the oncogenesis and development of hepatocellular carcinoma through the MAPK signaling pathway

**DOI:** 10.1002/mco2.510

**Published:** 2024-03-09

**Authors:** Yufu Peng, Jing Yu, Fei Liu, Leyi Tang, Bo Li, Wei Zhang, Kefei Chen, Haili Zhang, Yonggang Wei, Xuelei Ma, Hubing Shi

**Affiliations:** ^1^ Division of Liver Surgery Department of General Surgery West China Hospital Sichuan University Chengdu China; ^2^ Laboratory of Integrative Medicine Clinical Research Center for Breast State Key Laboratory of Biotherapy West China Hospital Sichuan University and Collaborative Innovation Center Chengdu China; ^3^ Department of Critical Care Medicine State Key Laboratory of Biotherapy and Cancer Center West China Hospital Sichuan University, China; ^4^ Department of Biotherapy West China Hospital and State Key Laboratory of Biotherapy Sichuan University Chengdu China

**Keywords:** growth and metastasis of liver cancer, Hepatocellular carcinoma, insulin‐like growth factor binding protein 3, microvascular invasion, TOX high mobility group box family member 3, tripartite motif containing 56

## Abstract

Microvascular invasion (MVI) has been widely valued in the field of liver surgery because MVI positivity indicates poor prognosis in hepatocellular carcinoma (HCC) patients. However, the potential molecular mechanism underlying the poor prognosis of MVI‐positive HCC patients is unclear. Therefore, this study focused on identifying the key genes leading to poor prognosis in patients with a high degree of malignancy of HCC by examining the molecular signaling pathways in MVI‐positive HCC patients. Through RNA sequencing, TOX high mobility group box family member 3 (TOX3) was demonstrated to be significantly highly expressed in MVI‐positive HCC tissues, which was associated with poor prognosis. The results of in vivo and in vitro showed that TOX3 can promote the oncogenesis and development of HCC by targeting key molecules of the MAPK and EMT signaling pathways. The IP‐MS results indicated that proteasome degradation of TOX3 in HCC cells is potentially mediated by a tripartite motif containing 56 (TRIM56, an E3 ligase) in HCC cells. Inhibiting TRIM56 enhances TOX3 protein levels. Overall, our study identified TOX3 as a key gene in the MAPK and EMT signaling pathways in HCC, and its overexpression confers significant proliferation and invasiveness to tumor cells.

## Graphical abstract

The depletion of the tripartite motif containing 56 in hepatocellular carcinoma (HCC) may potentiate the accumulation of TOX high mobility group box family member 3 (TOX3), and further regulate the classical signaling pathways of MAPK and EMT by increasing the expression level of insulin‐like growth factor binding protein 3, eventually promoting the growth and metastasis of HCCs.

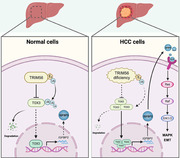

## 1 INTRODUCTION

Hepatocellular carcinoma (HCC) is the predominant primary hepatic malignant tumor with high postoperative recurrence rates and poor prognosis.^1^ Recently, microvascular invasion (MVI) has been widely valued in the field of liver surgery, because it occupies an important position among many adverse factors.^2^, ^3^, ^4^, ^5^ Previous studies have indicated that MVI serves as a precursor to both intrahepatic and extrahepatic metastasis of HCC, representing a significant manifestation of highly malignant HCC cells and signifying their heightened invasiveness. To date, the molecular mechanism underlying the poor prognosis of MVI‐positive HCC patients has not been determined. The recognition of key cancer‐promoting molecules could contribute to understanding the oncogenesis and development mechanism of HCC and identifying potential therapeutic targets.

The protein encoded by TOX high mobility group box family member 3 (TOX3) contains a high mobility group box.^6^ TOX3 may participate in the bending and releasing of DNA, as well as altering chromatin structure to promote transcription by binding to the eukaryotic cell promoters.^7^, ^8^, ^9^, ^10^ It can be regulated by binding phosphorylation proteins and can also play a role as a transcriptional costimulatory factor.^8^ However, the understanding of the role of TOX3 in tumors and its related mechanisms is in its infancy. According to the previously published literature, TOX3 can change the methylation status of its promoter and reduce the expression level of BRCA1, thus potentiating the growth and metastasis of breast cancer.^11^ Additionally, TOX3 can mediate the expression of the apoptosis‐related molecule RhoB and enhance the proliferation of colorectal tumors.^12^ Moreover, TOX3 can also strengthen the metastatic ability of lung cancer cells through regulating EMT and Hippo‐related signaling pathways.^13^

The tripartite motif containing 56 (TRIM56, an E3 ligase) is related to the degree of malignancy of liver, lung, hematological, and ovarian cancers. This motif inhibits the occurrence and metastasis of malignant tumors by targeting the degradation of certain carcinogenic proteins.^14^, ^15^, ^16^, ^17^ Yang et al. demonstrated that the TRIM56 expression level is down‐regulated in HCC tissues versus adjacent normal liver tissues, and the degree of downregulation of TRIM56 expression is closely related to tumor stage and prognosis. Mechanistically, TRIM56 can inhibit the oncogenesis and development of HCC by regulating the targeted degradation of key molecules in the Wnt signaling pathway.^14^ Yan et al. have shown that the binding of NEAT1 to the Wnt component DVL2 results in the interaction of the protein with TRIM56, which enhances the degradation of the DVL2 through the TRIM56‐mediated ubiquitin‐proteasome, thereby inhibiting the Wnt signaling pathway.^15^ Lu et al. indicated that TRIM56 can inhibit the invasion and development of lung cancer.^16^ Zhao et al. demonstrated that the downregulation of TRIM56 expression dramatically strengthens the invasive ability of ovarian malignant tumor cells, while overexpression of TRIM56 can reduce the invasive ability. Mechanically, TRIM56 can inhibit the invasive ability of malignant ovarian tumor cells by targeting Vimentin for degradation.^17^

In this study, high‐throughput RNA sequencing of HCC tissues revealed that TOX3 in MVI‐positive HCC tissues was highly expressed, thereby it was regarded as the focus molecule. Abnormal expression of TOX3 could strengthen oncogenesis and metastasis in HCC both in vitro and in vivo through the MAPK and EMT pathways, whereas proteasome degradation of TOX3 is potentially mediated by the E3 ligase TRIM56. This study demonstrated that TOX3 could represent a promising therapeutic target for addressing the oncogenesis and development of HCC. Thus, this study contributes to the understanding of the mechanisms underlying the oncogenesis and development of HCC and aids in the identification of potential therapeutic targets.

## 2 RESULTS

### 2.1 TOX3 in MVI‐positive HCC tissues was highly expressed, and negative prognosis in HCC patients was linked to TOX3 upregulation

**FIGURE 1:**
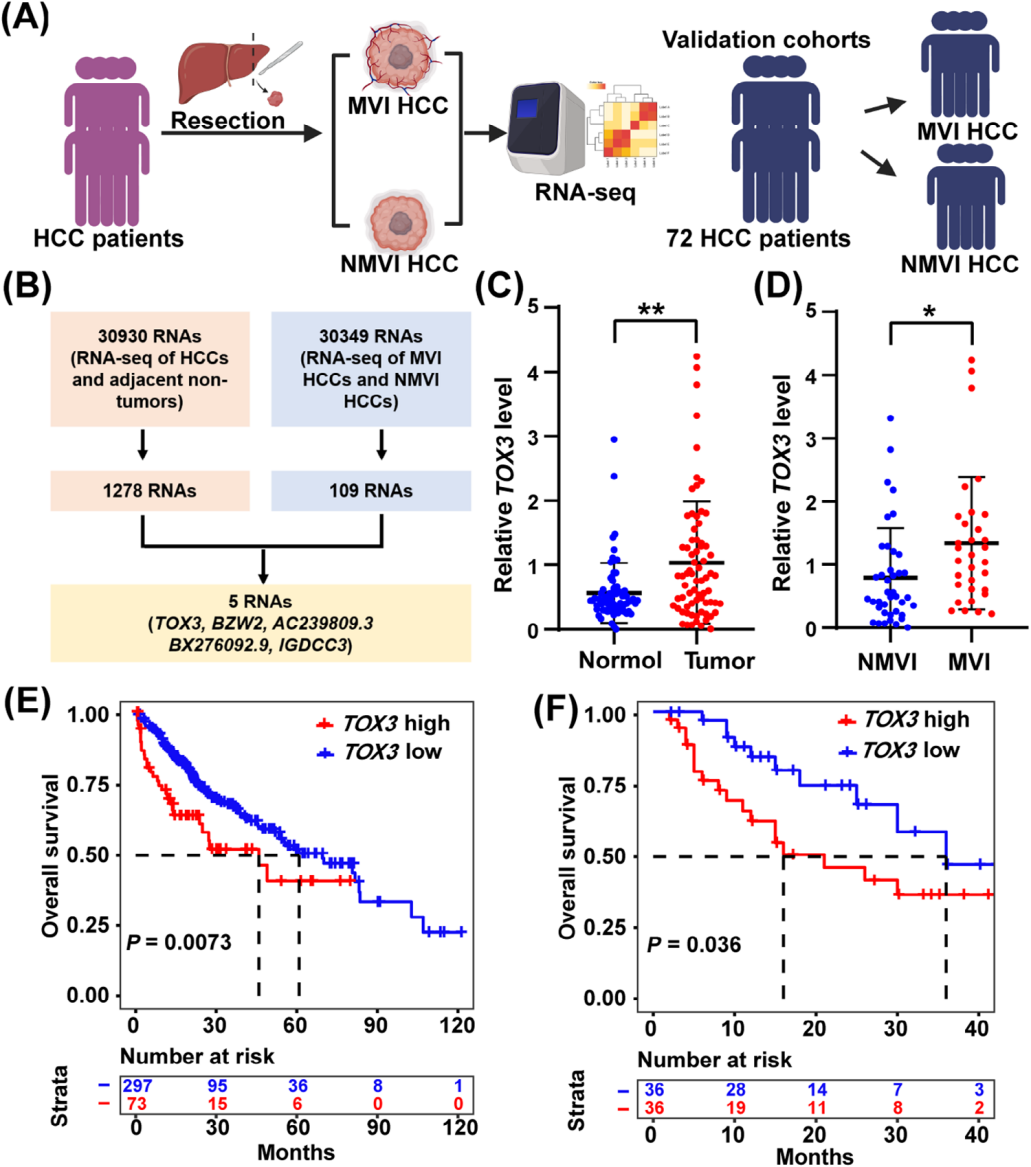
TOX high mobility group box family member 3 (TOX3) is upregulated in hepatocellular carcinoma (HCC) tissues with microvascular invasion (MVI) and associated with poor prognosis in HCC patients. (A) Schematic model presenting the process to identify and verify the target genes. (B) Flow diagram for selecting candidate genes through the intersection of tumor versus normal tissues and MVI versus NMVI HCC sequencing results. (C) The expression of *TOX3* in HCC tissues and adjacent normal tissues from 72 patients with HCC. (D) The above samples were divided into the HCC with MVI group (*n* = 32) and HCC without MVI group (*n* = 40). The difference in *TOX3* expression between the two groups was analyzed. (E) The OS of HCC patients with high *TOX3* expression versus low expression of HCC by TCGA database. (F) The OS of HCC patients with high *TOX3* expression versus low *TOX3* expression in 72 patients above. *p < 0.05, **p < 0.01, ***p < 0.001.

To explore the specific molecular mechanism underlying the poor prognosis caused by HCC with MVI, we collected 6 patients’ HCC tissues and paired adjacent normal liver tissues (three HCCs with MVI and three HCCs without MVI; Table S1). High‐throughput RNA sequencing was performed on a total of 12 tissue samples to identify and validate the target genes (Figure 1A). Based on the sequencing results above, the differentially expressed genes (DEGs) were further screened using specific statistical analysis conditions: the difference multiple fold change > 2 or < 0.5 and corrected *p‐*value (adj *p*) < 0.05. Figure 1B shows the screening process. The expression abundance of *TOX3* was consistent across adjacent normal tissue, HCC without MVI, and HCC with MVI tissues and showed a gradual increase among the three groups (Table S1). Additionally, the expression abundance of *TOX3* was high in tissue samples, and it was found that there were substantial differences in *TOX3* among the groups through the verification of the validation cohorts (Table S1 and Figure 1C,D). Thus, *TOX3* was ultimately selected as the focus of this study.

The present study examined *TOX3* expression in tumor and adjacent normal liver tissues from 72 HCC patients who underwent selected hepatectomy at our center. The validation results demonstrated that the *TOX3* expression level was markedly upregulated in cancer tissues versus adjacent normal liver tissues (Figure 1C). According to the pathological results of the above patients, we further divided them into an MVI‐positive HCC group (*n* = 32) and an MVI‐negative HCC group (*n* = 40). Subgroup analysis further demonstrated that *TOX3* in MVI‐positive HCC tissues was further highly expressed compared with that in the MVI‐negative HCC tissues (Table S2 and Figure 1D). Based on the statistical analysis of the TCGA database, patients with high *TOX3* expression had a worse prognosis (Figure 1E). Furthermore, real‐time quantitative polymerase chain reaction (RT‐qPCR) detection of HCC tissue was used to detect HCC tissue and assess the relationship between the *TOX3* expression level and patient survival rate. Patient groups were divided into two groups based on the median *TOX3* expression. Analysis of Kaplan‒Meier survival curves revealed a significantly poorer long‐term prognosis among HCC patients with high *TOX3* versus low *TOX3* expression (Figure 1F). Additionally, we further performed enrichment analysis (GO_BP gene terms) for the DEGs between HCC tissues and paired adjacent normal liver tissues (Figure S1A), and between the HCC patients in the MVI‐positive group and MVI‐negative group (Figure S1B), respectively. The results of enrichment analysis above mainly focused on cell proliferation, adhesion, and migration (Figure S1A,B).

### 2.2 TOX3 strengthens oncogenesis and development of HCC in vitro and in vivo

**FIGURE 2:**
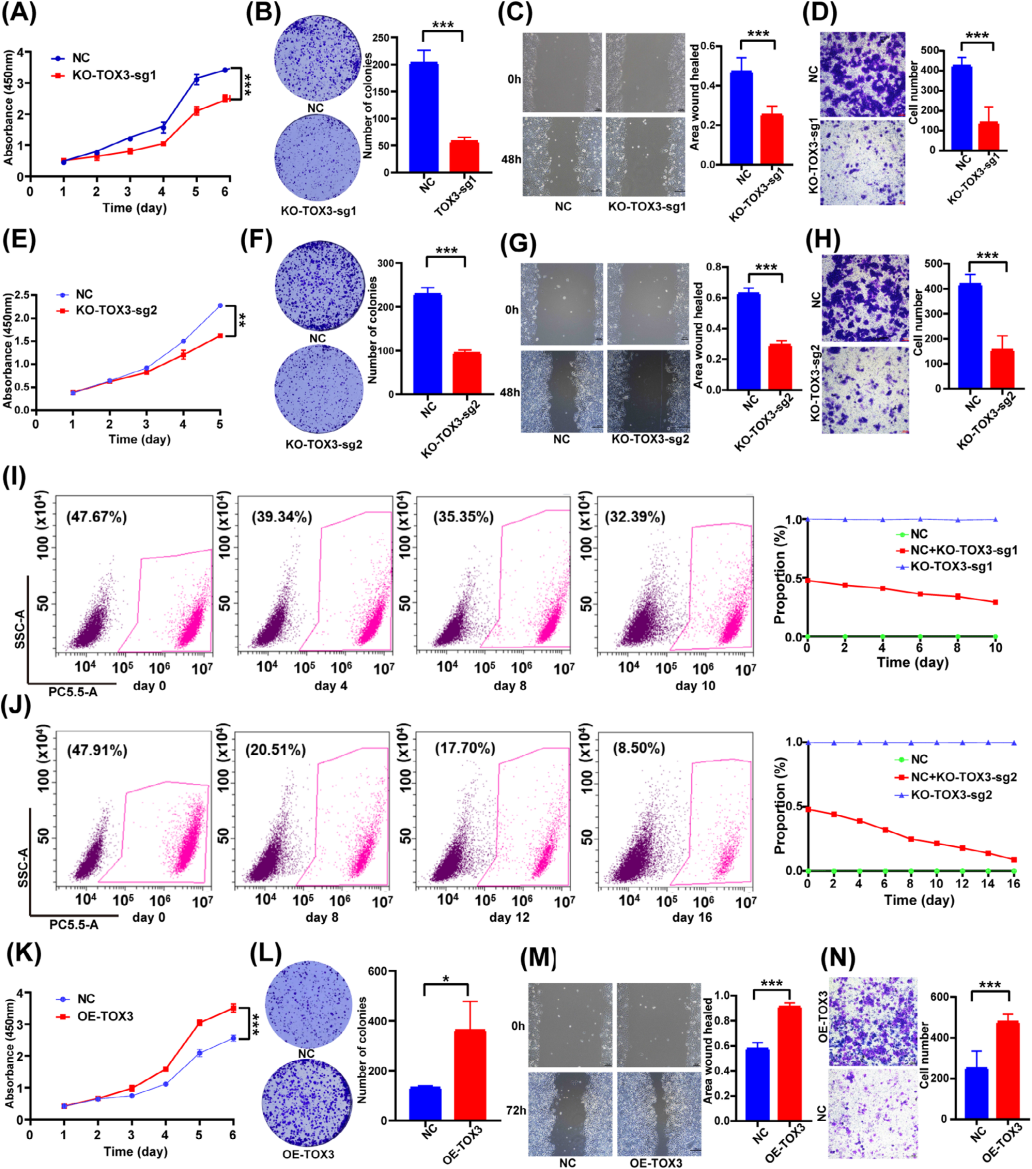
TOX high mobility group box family member 3 (TOX3) promotes the proliferation and migration of HCCLM3 cells in vitro. (A) and (E) After the TOX3 gene of HCCLM3 cells was knocked out by sgRNA1 and sgRNA2, respectively, the proliferation activity of the HCCLM3 cells was detected with a CCK‐8 kit. (B, F) The TOX3 gene of HCCLM3 cells was knocked out by sgRNA1 and sgRNA2, respectively, and the ability of colony formation was detected. (K, L) After overexpression of the TOX3 gene in HCCLM3, the proliferation capacity of cells was detected with a CCK‐8 kit, and the ability of colony formation was detected, respectively. (I, J) Coculturing WT cells (NC, no fluorescence) and HCCLM3 cells (mCherry, red fluorescence) of KO‐TOX3‐sgRNA1 and KO‐TOX3‐sgRNA2 respectively. The percentage of fluorescence positive rate was detected by flow cytometry. (C, G) After the TOX3 gene of HCCLM3 cells was knocked out by sgRNA1, Wound healing assay, and vitro cell migration assay were performed to detect the migratory capacity of the cells. (D, H) After the TOX3 gene of HCCLM3 was knocked out by sgRNA2, a wound healing assay, and vitro cell migration assay were performed to detect the migratory capacity of the cells. (M, N) After overexpression of the TOX3 gene in HCCLM3, wound healing assay and vitro cell migration assay were performed to detect the migratory capacity of the cells. *p < 0.05, **p < 0.01, ***p < 0.001.

TOX3 knockout in an HCC cell line (HCCLM3) was performed via the use of CRISPR/Cas9 (sgRNA1 and sgRNA2) to identify the role of TOX3 in the oncogenesis and development of HCC. The proliferation of HCCLM3 cells was suppressed after TOX3 gene knockout compared with that in the negative control group (the NC group) (Figure 2A,B,E,F). Moreover, a cell competition test between TOX3 knockout cells and control cells was performed, which showed that TOX3 knockout resulted in a decrease in the survival advantage of HCC cells (Figure 2I,J). Moreover, TOX3 knockout resulted in a substantial decrease in the migratory capacity of HCCLM3 cells. (Figure 2C,D,G,H). The overexpression of TOX3 in HCCLM3 cells induced by lentivirus transduction significantly enhanced the proliferation and migratory capacity of HCC cells (Figure 2K–N). To evaluate the antitumor effect of inhibiting Tox3 expression compared with that of classical targeted drugs, we used lenvatinib, which is the classical therapeutic target drug for HCC. The results indicated that, compared with that of lenvatinib, the inhibitory effect of TOX3 knockout on HCCLM3 cells was not inferior to the effect of lenvatinib on the IC_50_ (50 µM) against HCCLM3 (Figure S1C). To evaluate the degree to which TOX3 knockdown inhibited normal liver cell proliferation after TOX3 knockdown, we used shRNA to knock down TOX3 in HCCLM3 cells and normal liver cells (THLE‐2 cells). The results demonstrated that after knocking down TOX3, the proliferation capacity of HCCLM3 cells substantially decreased, but the proliferation ability of THLE‐2 cells was relatively unaffected (Figure S1D).

**FIGURE 3:**
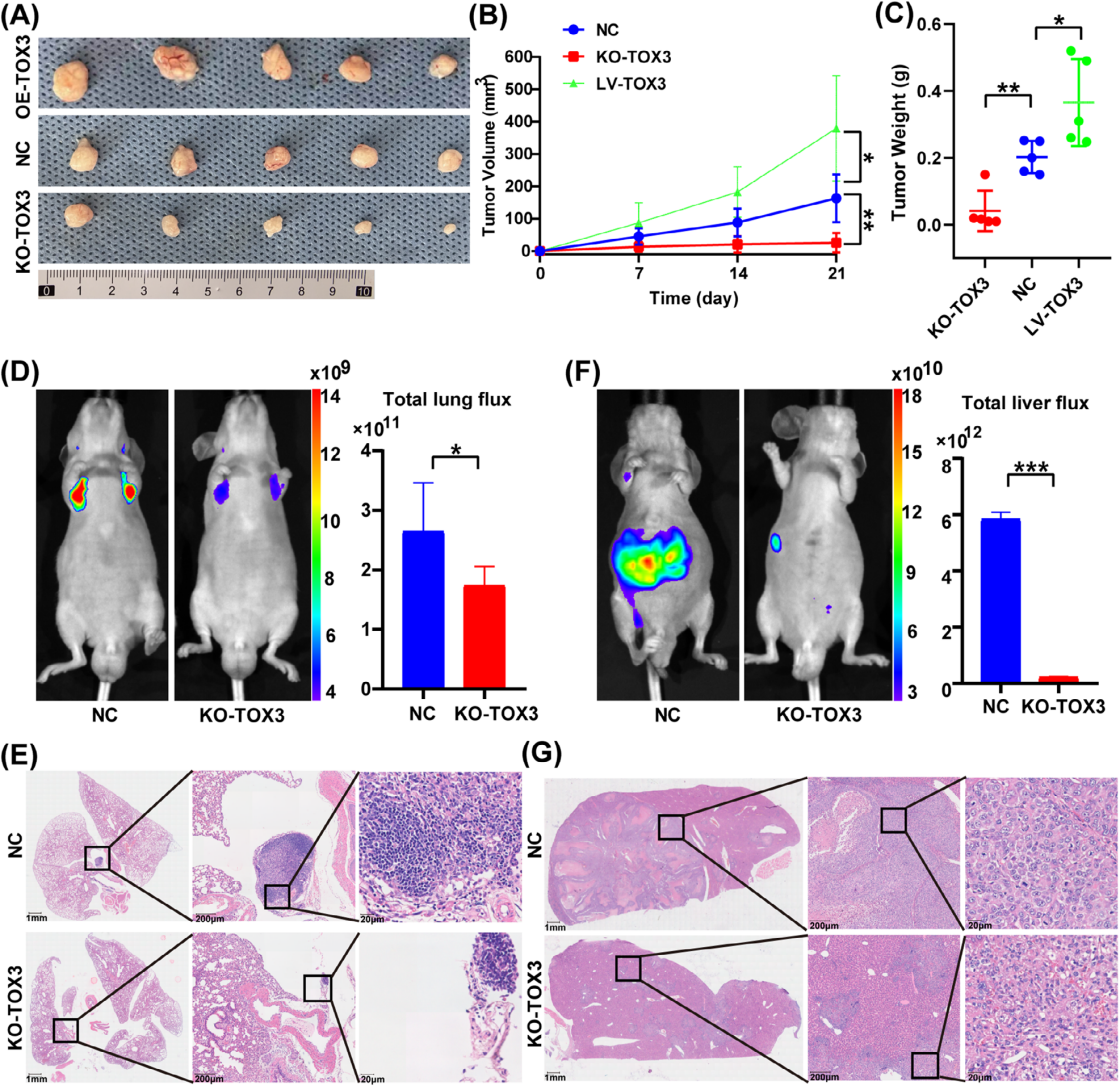
TOX high mobility group box family member 3 (TOX3) promotes the proliferation and migration ability of HCCLM3 cells in vivo. (A) The proliferative capacity of subcutaneous tumors in nude mice in the TOX3‐overexpression group, TOX3‐knockout group, and control group. (B) After the establishment of subcutaneous tumors, the tumor size of each group was measured weekly, and the tumor volume was calculated. (C) The subcutaneous tumor weights of each group. (D) For the establishment of the lung metastasis model, a small animal live imaging system chemiluminescence was performed to compare the fluorescence signals in the lung area of nude mice in the KO‐TOX3 group and NC group. (E) Lung metastasis was stained with H&E staining. (F) For the establishment of the liver orthotopic implantation model, a small animal live imaging system chemiluminescence was performed to compare the fluorescence signals in the abdominal area of nude mice in the KO‐TOX3 group and NC group. (G) Liver in situ tumors were stained with H&E staining. *p < 0.05, **p < 0.01, ***p < 0.001.

To explore whether the knockout or overexpression of the TOX3 gene affects the proliferative capacity of HCC cells in vivo, HCCLM3 cells (including the TOX3 knockout group, TOX3 overexpression group, and NC group) were used to establish the subcutaneous tumor model in nude mice. The proliferative capacity of subcutaneous tumors in nude mice substantially decreased after knockout of the TOX3 gene (KO‐TOX3) (Figure 3A), and the size and weight of the subcutaneous tumors substantially decreased (Figure 3B,C). Additionally, the proliferation capacity of the nude mice in the subcutaneous tumor model was substantially enhanced after TOX3 was overexpressed (OE‐TOX3) (Figure 3A), and the size and weight of the subcutaneous tumors increased significantly after TOX3 was overexpressed (Figure 3B,C). The lung metastasis model of HCC in nude mice was established by using HCCLM3 cells, and the results of small animal live imaging system chemiluminescence showed that the fluorescence signal in the lung area was substantially decreased after the TOX3 knockout (Figure 3D), and the lung metastases after the TOX3 knockout were also decreased (Figure 3E). The liver orthotopic implantation model of nude mice was established by using HCCLM3 cells. The results of small animal live imaging system chemiluminescence showed that the fluorescence signal was substantially decreased in the abdominal area of the nude mouse model after the TOX3 knockout (Figure 3F), and H&E staining of liver in situ tumor sections showed that the number of hepatic in situ tumors was also decreased after TOX3 knockout (Figure 3G).

### 2.3 TOX3 regulates HCC oncogenesis and development by targeting insulin‐like growth factor binding protein 3

**FIGURE 4:**
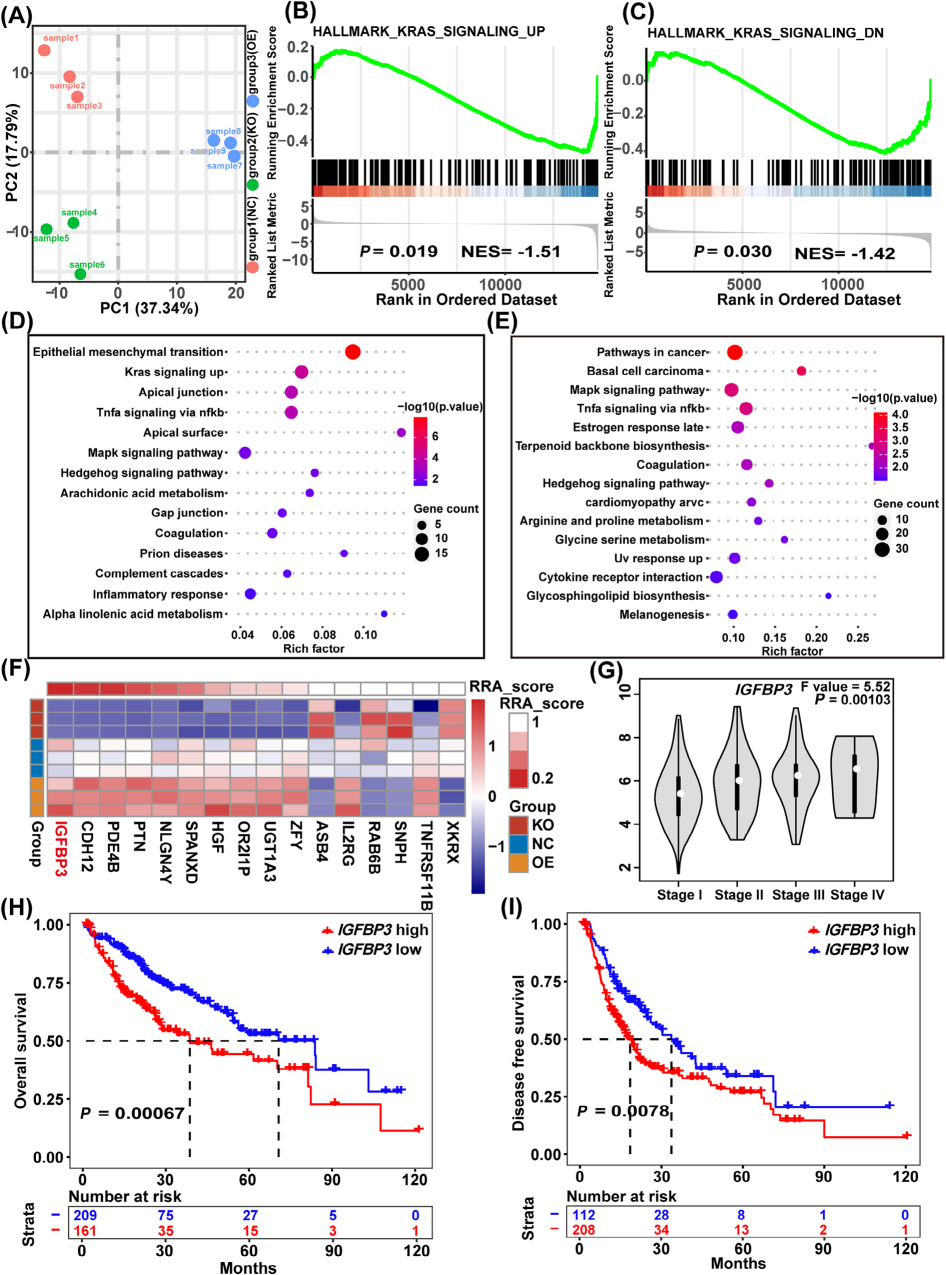
TOX high mobility group box family member 3 (TOX3) regulates signaling pathways involved in hepatocellular carcinoma (HCC) growth and metastasis. (A) PCA analysis of HCCLM3 cell lines in the TOX3 knockout group, overexpression group, and NC group were subjected. (B) and (C) GSEA showed that HALLMARK_KRAS_SIGNALING_UP and HALLMARK_KRAS_SIGNALING_DN pathways were down‐regulated in the TOX3 knockout and overexpression groups, respectively. (D) and (E) The results of the signaling pathway (KEGG and HALLMARK gene terms) enrichment analysis of differential expression genes of TOX3‐KO and TOX‐OE both showed that the regulation of TOX3 could significantly affect signaling pathways related to tumor growth and metastasis, such as MAPK and EMT. (F) The results of RRA analysis for the downstream target of TOX3. (G) Violin plot showing the relation of *IGFBP3* expression with the tumor stage of HCC. (H) and (I) The relationship between the *IGFBP3* expression with OS and DFS in HCC patients was analyzed by the TCGA database. *p < 0.05, **p < 0.01, ***p < 0.001.

To clarify the mechanism by which the TOX3 gene regulates the oncogenesis and metastasis capacity in HCC, HCCLM3 cell lines in the TOX3 knockout group, overexpression, and NC groups were subjected to high‐throughput RNA sequencing. The differential expression of genes in the three groups was consistent in each group, and there were significant differences among the three groups (Figure 4A). GSEA revealed that the HALLMARK_KRAS_SIGNALING_UP and HALLMARK_KRAS_SIGNALING_DN pathways were downregulated in the TOX3 knockout and overexpression groups, respectively (Figure 4B,C). Pathway enrichment analysis revealed that the regulation of TOX3 could significantly affect signaling pathways related to tumor growth and metastasis, such as the MAPK pathway and the EMT (Figure 4D,E). To further verify whether overexpression of TOX3 can directly upregulate the MAPK signaling pathway, we performed an enrichment analysis of GO_BP gene terms on genes that were statistically significant upregulation in the TOX3‐OE group. The MAPK pathway was significantly enriched, indicating that overexpression of TOX3 can upregulate the MAPK signaling pathway (Figure S1E). To identify the downstream target genes of TOX3, we screened for genes that exhibited significant upregulation in the KO‐TOX3 group while displaying significant down‐regulation in the OE‐TOX3 group; conversely, genes that were significantly downregulated in the KO‐TOX3 group but significantly upregulated in the OE‐TOX3 group were identified. This screening process yielded a total of 16 genes. Additionally, to pinpoint robust target genes, we employed the robust rank aggregation (RRA) algorithm to integrate the two sets of DEGs. Based on the RRA score, our analysis suggests that insulin‐like growth factor binding protein 3 (IGFBP3) may be the downstream target gene of TOX3 (Figure 4F). The sequencing results demonstrated that the level of *IGFBP3* was 0 after TOX3 knockout, and the level of *IGFBP3* increased after TOX3 was overexpressed (Figure S1F). This result was confirmed by RT‒qPCR (Figure S1G). Additionally, a Violin plot revealed that with the increase in IGFBP3 expression, the tumor stage was later (Figure 4G). TCGA analysis demonstrated that *IGFBP3* upregulation dramatically impacted HCC patient survival outcomes including the overall survival (OS) rate and tumor‐free survival rate (DFS) (Figure 4H,I).

**FIGURE 5:**
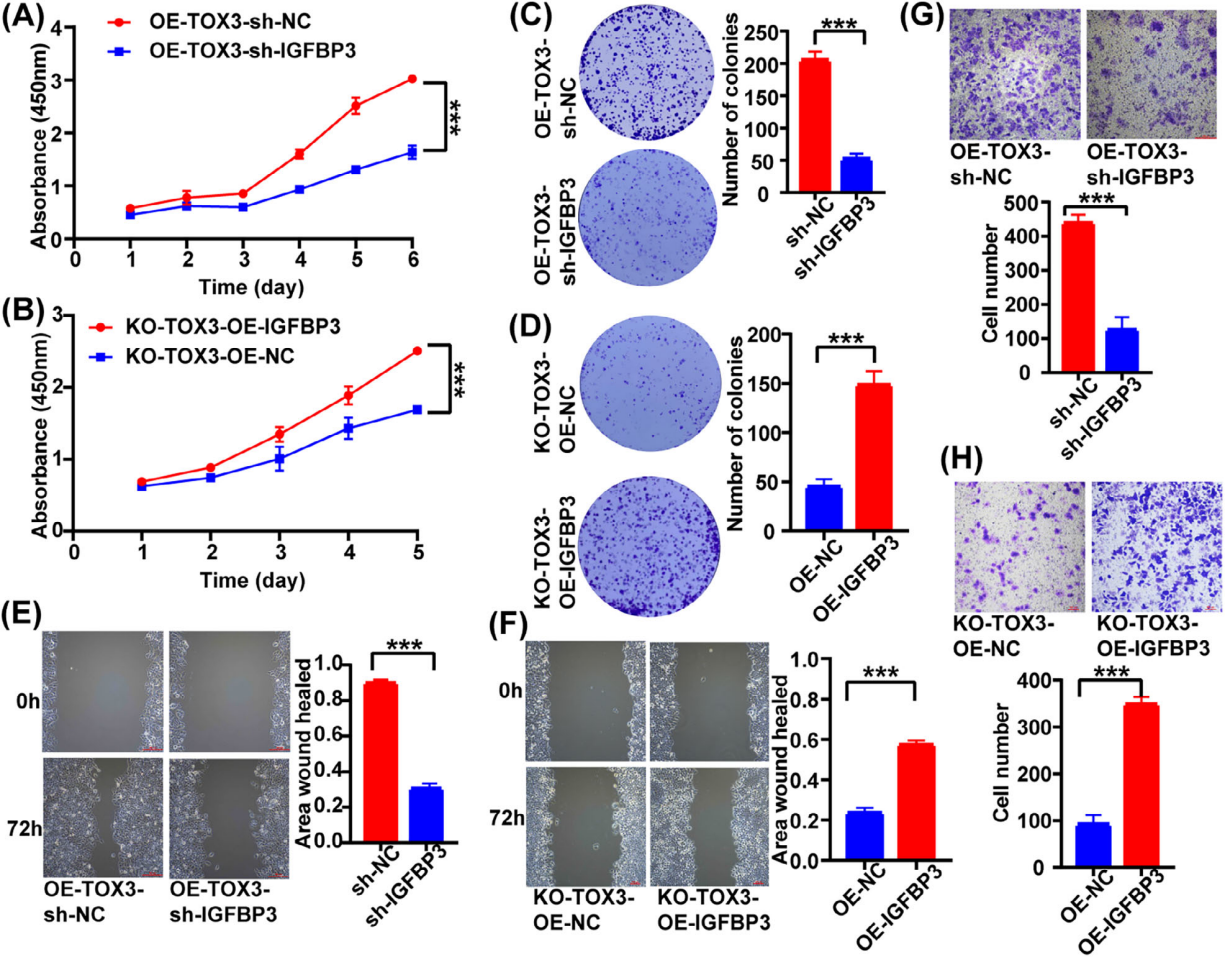
TOX high mobility group box family member 3 (TOX3) regulates the hepatocellular carcinoma (HCC) oncogenesis and development by targeting IGFBP3. (A) and (B) Knocking down IGFBP3 in TOX3‐OE HCCLM3 cells and overexpressing IGFBP3 in TOX3‐KO HCCLM3 cells, respectively, the proliferation activity of cells was detected with a CCK‐8 kit. (C) and (D) Knocking down IGFBP3 in TOX3‐OE HCCLM3 cells and overexpressing IGFBP3 in TOX3‐KO HCCLM3 cells, respectively, the ability of colony formation was detected. (E) and (F) Knocking down IGFBP3 in TOX3‐OE HCCLM3 cells and overexpressing IGFBP3 in TOX3‐KO HCCLM3 cells, respectively, wound healing assay was detected. (G) and (H) Knocking down IGFBP3 in TOX3‐OE HCCLM3 cells and overexpressing IGFBP3 in TOX3‐KO HCCLM3 cells, respectively, vitro cell migration assay was performed to detect the migratory capacity of the cells. *p < 0.05, **p < 0.01, ***p < 0.001.

To further clarify whether the regulatory effect of TOX3 on the malignant biological behavior of HCC cells depends on IGFBP3, we knocked down IGFBP3 in TOX3‐overexpressing HCCLM3 cells and overexpressed IGFBP3 in TOX3‐knockout HCCLM3 cells. According to our study, the oncogenesis and migratory capacity of HCCLM3 cells in the IGFBP3 knockdown group (OE‐TOX3‐sh‐IGFBP3) were substantially suppressed compared with the OE‐TOX3‐sh‐NC group (Figure 5A,C,E,G). In addition, compared with those in the NC group (KO‐TOX3‐OE‐NC), the oncogenesis and migratory capacity of HCCLM3 cells with suppressed proliferation and development after TOX3 knockout was restored by the overexpression of IGFBP3 in the experimental group (KO‐TOX3‐OE‐IGFBP3) (Figure 5B,D,F,H).

### 2.4 TOX3 regulates the key molecules of the MAPK and EMT signaling pathways by targeting IGFBP3

**FIGURE 6:**
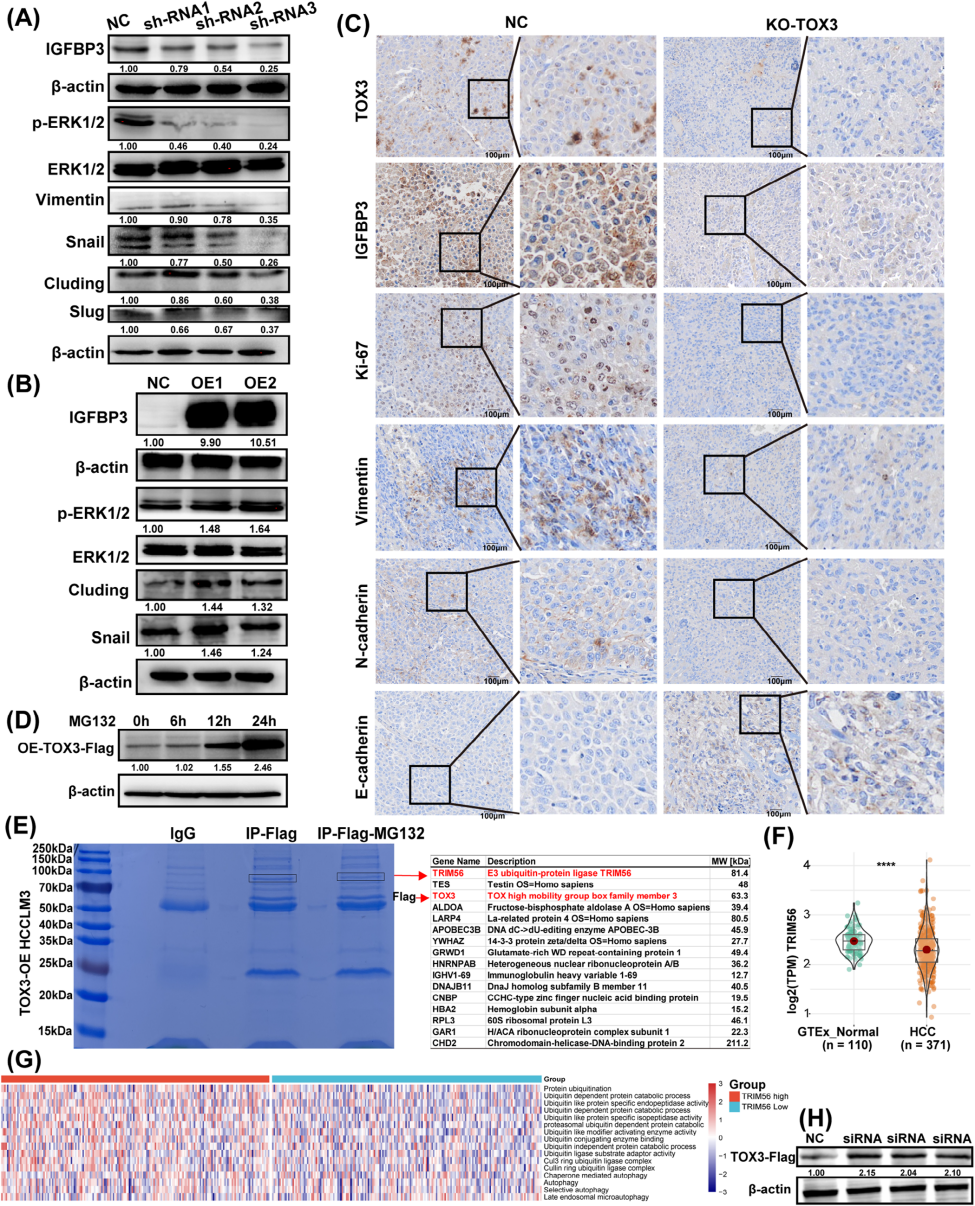
TOX high mobility group box family member 3 (TOX3) regulates key molecules in the MAPK and EMT signaling pathways by targeting IGFBP3, and the E3 ligase TRIM56 potentially mediates the proteasomal degradation of TOX3. (A) Effect of IGFBP3 knockdown on the expression of p‐ERK1/2, Vimentin, Snail, Cluding, and Slug in the MAPK and EMT pathways. (B) Effect of IGFBP3 overexpression on the expression of p‐ERK1/2, Snail, and Cluding in the MAPK and EMT pathways. (C) IHC staining about the expression of IGFBP3, Ki‐67, Vimentin, N‐cadherin, and E‐cadherin in the KO‐TOX3 and NC groups. (D) MG132 was added to the TOX3‐overexpressing HCCLM3 cells, and the TOX3‐FLAG expression was detected at 6, 12, and 24 h. (E) IP‐MS revealed the proteins interacting with TOX3 and explored the ability of the E3 ubiquitin ligase to target TOX3 degradation. (F) The expression of TRIM56 between the normal liver tissue (GETx) and hepatocellular carcinoma tissues (TCGA‐LIHC). (G) The heatmap of GSVA scores of the ubiquitin‐proteasome system and autophagy between TRIM56‐high and TRIM56‐low TCGA‐LIHC tumor samples, samples divided according to the median of the mRNA expression of TRIM56. Downregulation of TRIM56 significantly decreased the activity of the ubiquitin‐proteasome system but had no effect on autophagy. (H) TRIM56 was decreased and the expression of TOX3 was detected. *p < 0.05, **p < 0.01, ***p < 0.001.

The results of high‐throughput RNA sequencing showed that the regulatory effect of TOX3 on the malignant biological behavior of HCC cells involved mainly focused on signaling pathways related to tumor growth and metastasis, such as the KRAS, MAPK, and EMT pathways (Figure 4D,E and Figure S1E). In addition, a series of high‐quality related studies have shown that IGFBP3 can regulate signaling pathways related to proliferation and metastasis, including the MAPK, PI3K‐Akt, and EMT pathways.^18^, ^19^, ^20^, ^21^ Thus, western blot (WB) was employed to clarify whether IGFBP3, which is regulated by TOX3, can affect the expression of key molecules in the MAPK and EMT pathways. The results indicated that the knockdown of IGFBP3 (OE‐TOX3‐sh‐IGFBP3 HCCLM3) led to a significant decrease in the key proteins p‐ERK1/2, vimentin, Snail, Cluding, and Slug in the MAPK and EMT pathways (Figure 6A). Additionally, IGFBP3 upregulation substantially increased the expression of p‐ERK1/2, Snail, and Cluding (Figure 6B). Additionally, immunohistochemical (IHC) staining indicated that TOX3 knockout substantially downregulated IGFBP3, Ki‐67, vimentin, and N‐cadherin expression but upregulated E‐cadherin expression (Figure 6C). These experimental results are consistent with previous high‐quality studies, indicating that IGFBP3 can target key molecules involved in regulating MAPK and EMT signaling pathways.

### 2.5 Proteasome degradation of TOX3 is potentially mediated by the E3 ligase TRIM56

During this experiment, we found that there was a dramatic difference in the level of TOX3 transcription and protein expression. To explore the posttranslational ubiquitination of TOX3, we tried to intervene in the targeted degradation process of the intracellular proteasome system by using the proteasome inhibitor MG132. Consequently, TOX3 was upregulated after MG132 intervention and substantially increased with the prolongation of MG132 treatment in TOX3‐overexpressing HCCLM3 cells (Figure 6D). These results suggest that the TOX3 protein undergoes intracellular degradation via the ubiquitin‐proteasome system. To further investigate the specific ubiquitin ligase responsible for targeting the degradation of TOX3, we sequenced the protein spectrum of immunoprecipitated proteins by using the protein complex combined with Flag to pull down TOX3 in TOX3 overexpressing HCCLM3 cells. The sequencing results of IP‐MS showed that the TRIM56 potentially participated in the targeted proteasomal degradation of TOX3 (Figure 6E). Furthermore, we further analyzed the expression level of TRIM56 in the normal liver tissue (GETx) and HCC tissue (TCGA‐LIHC) samples, and the results indicated that the expression level of TRIM56 in HCC tissues was markedly down‐regulated than that in the normal liver tissues (Figure 6F). Heatmap of GSVA scores of the autophagy and ubiquitin‐proteasome systems between TRIM56‐high and TRIM56‐low TCGA‐LIHC tumor samples. Samples were divided according to the median of the expression level of TRIM56. TRIM56 downregulation significantly decreased the activity of the ubiquitin‐proteasome system but had no effect on autophagy (Figure 6G and Figure S2A). Finally, small interfering RNA (siRNA) was used to silence TRIM56 in OE‐TOX3‐FLAG HCCLM3 cells, which demonstrated that the downregulation of TRIM56 can increase the expression level of TOX3 (Figure 6H). This part of the experimental results preliminarily explored the upstream regulatory mechanism of TOX3, and the results showed that proteasomal degradation of TOX3 is potentially mediated by the E3 ligase TRIM56.

## 3 DISCUSSION

The degree of malignancy of HCC is high, resulting in a poor prognosis for patients, and the underlying mechanisms of hepatocarcinogenesis and development remain incompletely understood. At present, much‐published literature has demonstrated that MVI positivity indicates poor prognosis in HCC patients and it occupies an important position among many adverse factors.^2^, ^3^, ^4^, ^22^ Previous studies have shown that MVI serves as a precursor to both intrahepatic and extrahepatic metastasis of HCC, representing a significant manifestation of highly malignant HCC cells and signifying their heightened invasiveness.^23^ However, the molecular mechanism contributing to the poor prognosis in MVI‐positive HCC patients remains unclear. Consequently, MVI was used as the entry point for identifying highly malignant markers of HCC to determine the key genes that could lead to poor prognosis in patients with a high malignancy degree of HCC. HCC tissues from patients with MVI, HCC tissues without MVI, and adjacent normal tissues were sequenced, and the key target gene *TOX3* was screened out after statistical analysis. The results of expanded sample size verification and a series of in vitro and in vivo functional phenotyping experiments demonstrated that TOX3 significantly promoted the growth and metastasis of HCC.

Previous studies have shown that the proteins encoded by *TOX3* may participate in the bending and release of DNA and changes in chromatin structure. Furthermore, other published studies have indicated that TOX3 can act as a transcriptional costimulatory factor, which can promote the transcription of downstream genes.^7^, ^8^, ^9^, ^10^, ^24^ The sequencing results of the TOX3 overexpression group, knockout group, and NC group showed that TOX3 could regulate many biological processes (BPs) and molecular‐related functions (MFs) related to cell proliferation and metastasis. These include but are not limited to cell proliferation, angiogenesis, cell adhesion, cell connection, extracellular matrix construction, or growth factor activity. Considering the phenotypes observed in the aforementioned cells and animal models, it can be inferred that TOX3 enhances the malignant biological behavior of HCC cells by affecting cell proliferation, exfoliation, and metastasis. Moreover, our enrichment analysis demonstrated that the regulation of TOX3 could significantly affect classical signaling pathways related to KRAS, MAPK, EMT, etc. In addition, IGFBP3 was identified as a downstream target. According to previous studies, TOX3 can promote the oncogenesis and development of breast cancer, colorectal cancer, and lung cancer through the MAPK, EMT, and Hippo‐related signaling pathways.^12^, ^13^ Thus, the differential gene enrichment analysis of our study is relatively reliable. Interestingly, based on previous studies, IGFBP3 targets the regulation of classical signaling pathways and key molecules such as MAPK, EGFR, AKT, and EMT to regulate tumor proliferation and metastasis.^18^, ^19^, ^20^, ^21^ IGFBP3 is the main member of the IGFBP family, especially IGFBP3 secreted into the circulatory system occupies an important position.^18^ Much literature has reported that IGFBP3 can regulate cell proliferation and migratory capacity by binding to cell surface receptors and stimulating its activity.^25^, ^26^, ^27^ Song et al. showed that galectin‐3 can regulate the expression of IGFBP3 and vimentin through β‐catenin and substantially strengthen the angiogenesis and the development of HCC, thereby IGFBP3 markedly upregulates the proliferation and migration of HCC cells.^20^ Janet et al. have also indicated that IGFBP3 enhances the oncogenesis and development of breast cancer; mechanically, it can upregulate the EGFR phosphorylation and activate the p44/42 and p38 MAPK classical signaling pathways.^19^ Deobrat et al. demonstrated that the M6A reader YTHDF2 can upregulate IGFBP3 expression through MYC, which subsequently modulates the AKT and EGFR through IGFBP3 to enhance the growth of glioma. Notably, the overexpression of IGFBP3 can restore the tumorigenesis of glioma caused by YTHDF2 deletion in vivo.^28^ Liu et al. showed that IGFBP3 can directly regulate the key molecules of the AKT, EMT, and MAPK signaling pathways, thereby influencing the oncogenesis and development of renal carcinoma.^29^ Therefore, according to previous studies, pathway enrichment analysis, WB assays, and IHC staining in this study, it is plausible to suggest that TOX3 up‐regulates IGFBP3 expression level by binding to the CRE site in the promoter of the downstream gene IGFBP3. Thus, it promotes the malignant biological behavior of HCC by regulating the classical pathway of tumor growth and metastasis. Moreover, according to the results of this study, the inhibitory effect of downregulating Tox3 expression on the proliferative capacity of HCC tumor cells was dramatically greater than that on human normal hepatocytes. Therefore, HCC‐targeted therapeutic drugs could be further developed from TOX3, especially for highly malignant HCC patients with MVI positivity, which may improve the survival rate of these high‐risk patients with recurrence.

**FIGURE 7:**
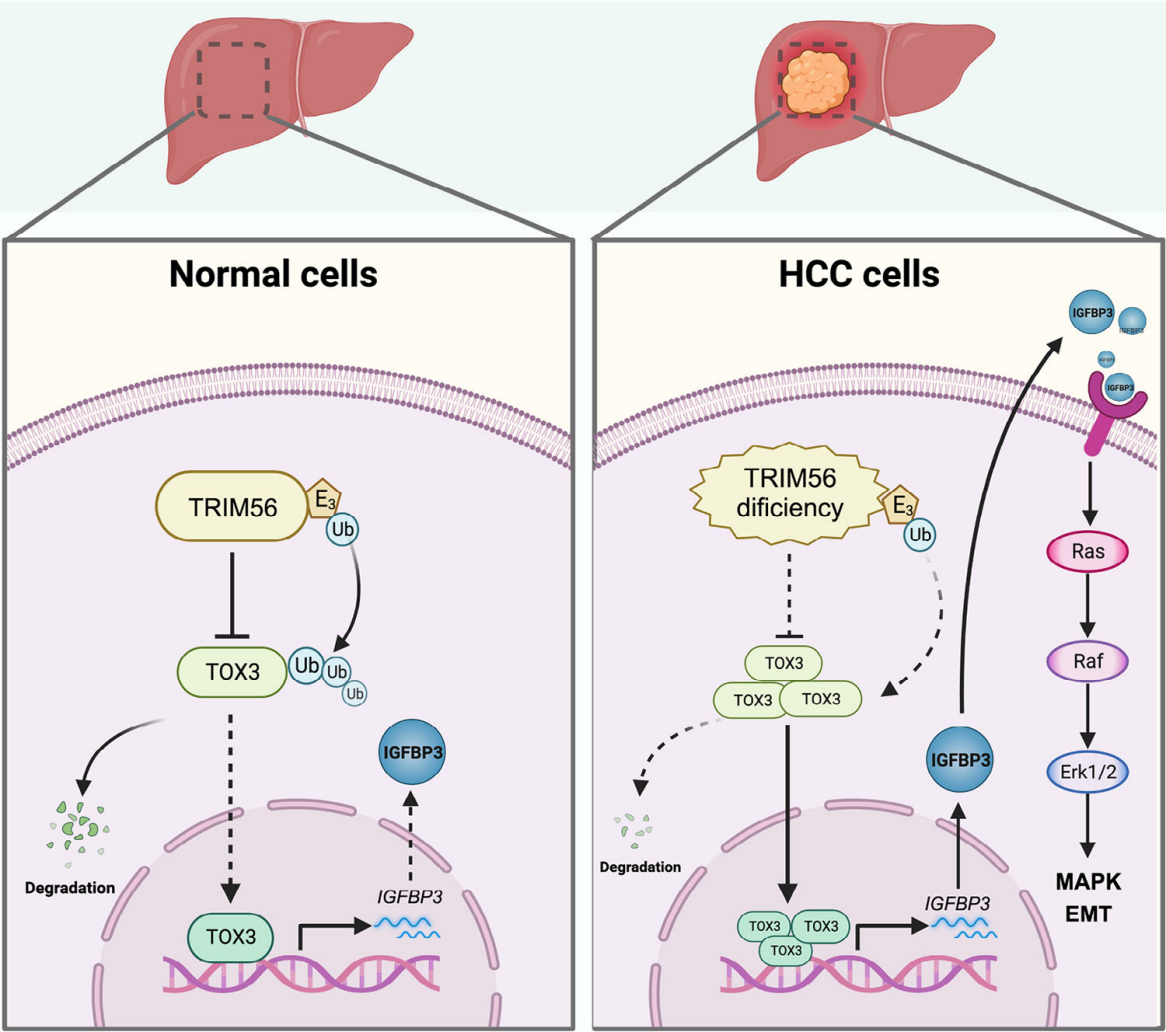
Schematic model presenting the mechanism by which TOX high mobility group box family member 3 (TOX3) promotes the oncogenesis and development of hepatocellular carcinoma (HCC) cells.

For the regulation of the upstream molecular mechanism of TOX3, this study demonstrated that TOX3 potentially undergoes posttranslational ubiquitin modification and undergoes intracellular degradation through the ubiquitin‐proteasome system. However, E3 ubiquitin ligases are critical for the intracellular degradation of substrate proteins in the proteasome. Additionally, many published literature have indicated that the E3 ligase is related to tumorigenesis and tumor development.^30^, ^31^, ^32^, ^33^ The IP‐MS results showed that TRIM56 potentially participated in the targeted proteasomal degradation of TOX3. Subsequent employment use of siRNA to knock down TRIM56 resulted in an increase in the expression of TOX3, leading to the conclusion that proteasomal degradation of TOX3 is potentially mediated by the E3 ligase TRIM56. Notably, some published literature has demonstrated that TRIM56 can target and degrade some oncoproteins, thereby inhibiting the occurrence and development of malignant tumors.^14^, ^15^, ^16^, ^17^ Yang et al. showed that the expression level of TRIM56 in HCC tissues is downregulated compared with adjacent normal liver tissues, and the downregulation degree of TRIM56 expression is closely related to tumor stage and prognosis. Mechanistically, TRIM56 can inhibit the oncogenesis and metastasis of HCC by targeting the degradation of proteins related to the Wnt signaling pathway.^14^ Therefore, the depletion of TRIM56 in HCC may potentiate the accumulation of TOX3 and further regulate the classical signaling pathways of MAPK and EMT by upregulating the IGFBP3 expression level, eventually promoting the oncogenesis and development of HCC (Figure 7).

## 4 CONCLUSIONS

In summary, TOX3 in HCC tissues was upregulated, and compared with MVI‐negative HCC tissues, TOX3 expression level in MVI‐positive HCC tissues was markedly further increased. TOX3 overexpression was associated with a more malignant degree of HCC and negative prognosis in HCC patients. TOX3 up‐regulates IGFBP3 expression level and fosters the oncogenesis and development of HCC via the MAPK and EMT pathways. Additionally, the E3 ligase TRIM56 potentially mediates the proteasomal degradation of TOX3. The above studies could lead to the identification of potential therapeutic targets for HCC treatment.

## 5 MATERIALS AND METHODS

All clinical tissue specimens and related clinical information involved in this study were in accordance with the principles of the Declaration of Helsinki. The animal experiment scheme was approved by the Animal Ethics Committee of our hospital (20220302033).

### 5.1 Clinical samples

The specimens of the sequencing group (including 12 MVI‐positive and ‐negative HCC and paired liver normal tissues) were collected from the operating room of our center (Table S1), and the samples of the validation group were collected from the biological sample bank of our hospital (Table S2), which included HCC patients received selective curative hepatectomy from 2018 to 2019 (2021‐DI‐009). The inclusion criteria were pathological diagnosis of HCC and no adjuvant treatment before the operation. The exclusion criteria were patients combined with another type of cancer, and the patients’ data were incomplete.

### 5.2 Cell culture

The HCC cells HCCLM3 and the Human liver immortalized cells THLE‐2 involved are all human cell lines commonly used. HCCLM3 cells were purchased from the cell bank of the Shanghai Institute of Life Sciences, and 293T cells were purchased from the cell bank of Shanghai Type Culture Collection (Chinese Academy of Sciences). THLE‐2 cells were purchased from Wuhan Procell Life Science & Technology Co. 293T and HCCLM3 cells were cultured in 90% DMEM high glucose medium supplemented with 10% fetal bovine serum (FBS) and 1% penicillin/streptomycin. THLE‐2 cells were cultured in a Complete medium of THLE‐2 including BEGM and 10% FBS.

### 5.3 Animals

The animals used in the animal experiment were BALB/Nude male mice, which were purchased from Huafukang Company and Gempharmatech Company.

### 5.4 Single‐cell clone selection

We used the CRISPR‐cas9 system to obtain complete knockout clones of TOX3 by transfecting lentivirus for CRISPR targeting of the TOX3 gene into HCC cells. Two target sequences including TAACTATATGAATATGGCTG (sgRNA1) and CAGTACATCCGGCATGCCTA (sgRNA2), were used for TOX3. Stably transfected cells were selected using puromycin and single‐cell clones were then isolated using a flow cytometer.

### 5.5 CCK‐8

Approximately 1.6×103 cells were suspended and added to each well of a 96‐well plate. A CCK‐8 proliferation detection kit from the MCE Company was used to detect cell proliferation. The absorbance OD value of the cells was detected by an EonTM Microplate Reader with a wavelength of 450 nm.

### 5.6 Colony formation

We seeded 2000 cells per well in a 6‐well plate and allowed them to incubate for 2 weeks. After fixation with 4% paraformaldehyde, we stained the colonies with 0.1% crystal violet.

### 5.7 Wound healing assay

We seeded cells into a 6‐well plate and created scratches with a 10 µL pipette tip. The rate of cell migration was recorded under a microscope, and the location of mapping at each time point was as consistent as possible.

### 5.8 In vitro cell migration assay

We seeded 5×104 cells in the upper Transwell chamber with an 8 µm pore size. The inner chamber contained serum‐free DMEM, while 600 µL of DMEM containing 10% FBS was added to each lower chamber. After 48 h, we fixed and stained the cells on the lower surface of the chamber with 4% paraformaldehyde and 0.1% crystal violet respectively, then recorded the images.

### 5.9 Cell competition test

TOX3‐KO cells and negative control (NC) cells were cocultured.^34^ TOX3‐KO cells expressed the red fluorescent protein mCherry, while the NC cells had no fluorescence. 1×10^6^ TOX3‐KO and NC cells were obtained, respectively; a total of 2×106 cells were fully mixed in the same aseptic centrifuge tube. 1/3 of the cells above was used to detect the ratio of red fluorescent cells (TOX3‐KO) by flow cytometry, and the remaining cells were transferred into triplicate wells of a 6‐well plate for coculture. Additionally, the cocultured mixed cells were detected by flow cytometry at an interval of one day, the percentage of fluorescence positive rate was analyzed, and the above operation was repeated.

### 5.10 Protein immunoprecipitation combined with mass spectrometry

Immunoprecipitation (IP) antibody Flag was added to the target protein and then flipped overnight at 4°C for at least 16 h. The complex was added to the pretreated magnetic beads (Protein A/G, MCE) to incubate with flipping and binding. The mixture was placed on a magnetic frame, and IP lysis buffer was used to wash the magnetic beads, then the proteins were denatured and the magnetic beads were separated under the setting conditions of 100°C and 10 min. The tube was placed on the magnetic frame, and the pull‐down complex protein was collected. The IP proteins were subjected to electrophoresis and sent for mass spectrometry sequencing analysis.

### 5.11 Animal experiment

We used five‐week‐old male BALB/c nude mice established tumor models. In detail, the subcutaneous tumor model of liver cancer, the liver orthotopic implantation model, and the lung metastasis model were established with different numbers of HCC cells. 2 × 106 HCC cells within 100 µL suspension were established the subcutaneous tumor model by injecting subcutaneously HCC cells into the right chest and abdomen of nude mice, and a semicircular colliculus could be formed locally. The volume of the subcutaneous tumor and the final tumor weight were recorded. 1×106 HCC cells within 100 µL suspension were established the liver orthotopic implantation model by injecting into the liver of nude mice, and the red fluorescence from mCherry in the HCC cell line was strong; thus, the fluorescence technique of small animal live imaging systems could be selected for detection and recording. 1.5 × 106 HCC cells within 125 µL suspensions were established in the lung metastasis model by injecting intravenously into the tail of nude mice, and the tumor metastases in vivo would be recorded by small animal live imaging system chemiluminescence mode.

### 5.12 RNA sequencing data analysis

First, we used TrimGalore (v0.6.7) to perform quality trimming on the raw sequencing data. Subsequently, we aligned the processed clean reads to the human genome (GRCh38) using Kallsito (v0.46.1) to obtain the gene expression matrix. Following this, we employ DESeq2 (v1.38.3) software for differential expression analysis, with the criteria for selecting differentially expressed genes as follows: (1) absolute value of log2 (fold change) > 1; (2) adjusted *p*‐value < 0.05. Both GSEA analysis and pathway enrichment analysis of differentially expressed genes are conducted using clusterProfiler (v4.6.0), the KEGG, HALLMARK, and GO‐BP gene terms used for analysis were downloaded from MSigDB (https://www.gsea‐msigdb.org/gsea/msigdb). Enrichment of up‐regulated genes in the TOX3‐OE group was performed in the KOBAS‐i database (http://bioinfo.org/kobas/). RobustRankAggreg (v1.2.1) was used for integrating differential expression genes. Gene terms related to the ubiquitin‐proteasome system and autophagy were downloaded from the MSigDB database, and the molecular pathway activity of tumor samples in the TCGA‐LIHC dataset was quantified via GSVA (v1.46.0).

The mRNA expression matrix of the TCGA‐LIHC dataset and its corresponding clinical information are obtained from UCSC Xena (https://xena.ucsc.edu/). The mRNA expression matrix of normal liver tissue was obtained from the GTEx dataset and can be also downloaded from UCSC Xena.

### 5.13 Statistical analysis

The data of this study was analyzed by GraphPad Prism 8.0, ImageJ, and ipwin 32. The schematic diagram of this paper is completed by using the BioRender online drawing website. Student's T‐test was used for the analysis of the continuous variables, and the Pearson chi‐square test or Fisher exact probability method was used for the analysis of the counting data. The survival rate of patients with liver cancer was statistically analyzed by the Kaplan‒Meier method, and the difference in survival rate was compared by the log‐rank test. *p*‐Values < 0.05 were considered significant.

## AUTHOR CONTRIBUTIONS

Designed this work (L.F., M.X.L, W.Y.G., and S.H.B.). Analyzed this data (P.Y.F. and Y.J.). Wrote this paper (P.Y.F. and Y.J.). Revised this paper (P.Y.F., Y.J., T.L.Y., L.B., L.F., W.Y.G., M.X.L., and S.H.B.). Took part in some major experiments (P.Y.F., Y.J., C.K.F, Z.H.L., Z.W., and T.L.Y). All authors have read and approved the final manuscript.

## CONFLICT OF INTEREST STATEMENT

The authors declare no conflict of interest.

## ETHICS STATEMENT

The clinical tissue specimens’ acquisition was approved by the biological sample bank of West China Hospital of Sichuan University (2021‐DI‐009). The animal experiment scheme was approved by the Animal Ethics Committee of West China Hospital of Sichuan University (20220302033).

## Supporting information

Supporting Information

## Data Availability

All data supporting the results of this study are available from the corresponding author according to rationality.
