# Supplementary material for: Accumulation of TOX high mobility group box family member 3 promotes the oncogenesis and development of hepatocellular carcinoma through the MAPK signaling pathway
Source: MedComm (2020). 2024 Mar 9;5(3):e510. doi: 10.1002/mco2.510 (PMC10924639; doi:10.1002/mco2.510)
Supplement: Supplementary file 1 — Supporting Information [file MCO2-5-e510-s001.docx]

**Title**: Accumulation of TOX high mobility group box family member 3 promotes the oncogenesis and development of hepatocellular carcinoma through MAPK signaling pathway

**Authors**: Yufu Peng^1,2^, Jing Yu^2^, Fei Liu^1^, Leyi Tang^2^, Bo Li^1^, Wei Zhang^3^, Kefei Chen^1^, Haili Zhang^1^, Yonggang Wei^4^, Xuelei Ma^5^, Hubing Shi^6^

**Authors' affiliations**: ^1^Division of Liver Surgery, Department of General Surgery, West China Hospital, Sichuan University, Chengdu, China

^2^Laboratory of Integrative Medicine, Clinical Research Center for Breast, State Key Laboratory of Biotherapy, West China Hospital, Sichuan University and Collaborative Innovation Center, Chengdu, China.

^3^Department of Critical Care Medicine, State Key Laboratory of Biotherapy and Cancer Center, West China Hospital, Sichuan University, China

^4^Division of Liver Surgery, Department of General Surgery, West China Hospital, Sichuan University, Chengdu, Sichuan 610041, China. Electronic address: weiyonggang@wchscu.edu.cn

^5^Department of Biotherapy, West China Hospital and State Key Laboratory of Biotherapy, Sichuan University, Chengdu, Sichuan 610041, China. Electronic address: drmaxuelei@gmail.com.

^6^Laboratory of Integrative Medicine, Clinical Research Center for Breast, State Key Laboratory of Biotherapy, West China Hospital, Sichuan University and Collaborative Innovation Center, Chengdu, Sichuan 610041, China. Electronic address: shihb@scu.edu.cn.

**First author**: Yufu Peng, Jing Yu, Fei Liu, and Leyi Tang contributed equally.

**Corresponding authors**: Yonggang Wei, Xuelei Ma and Hubing Shi

**Figures**

**Figure S1:**

**
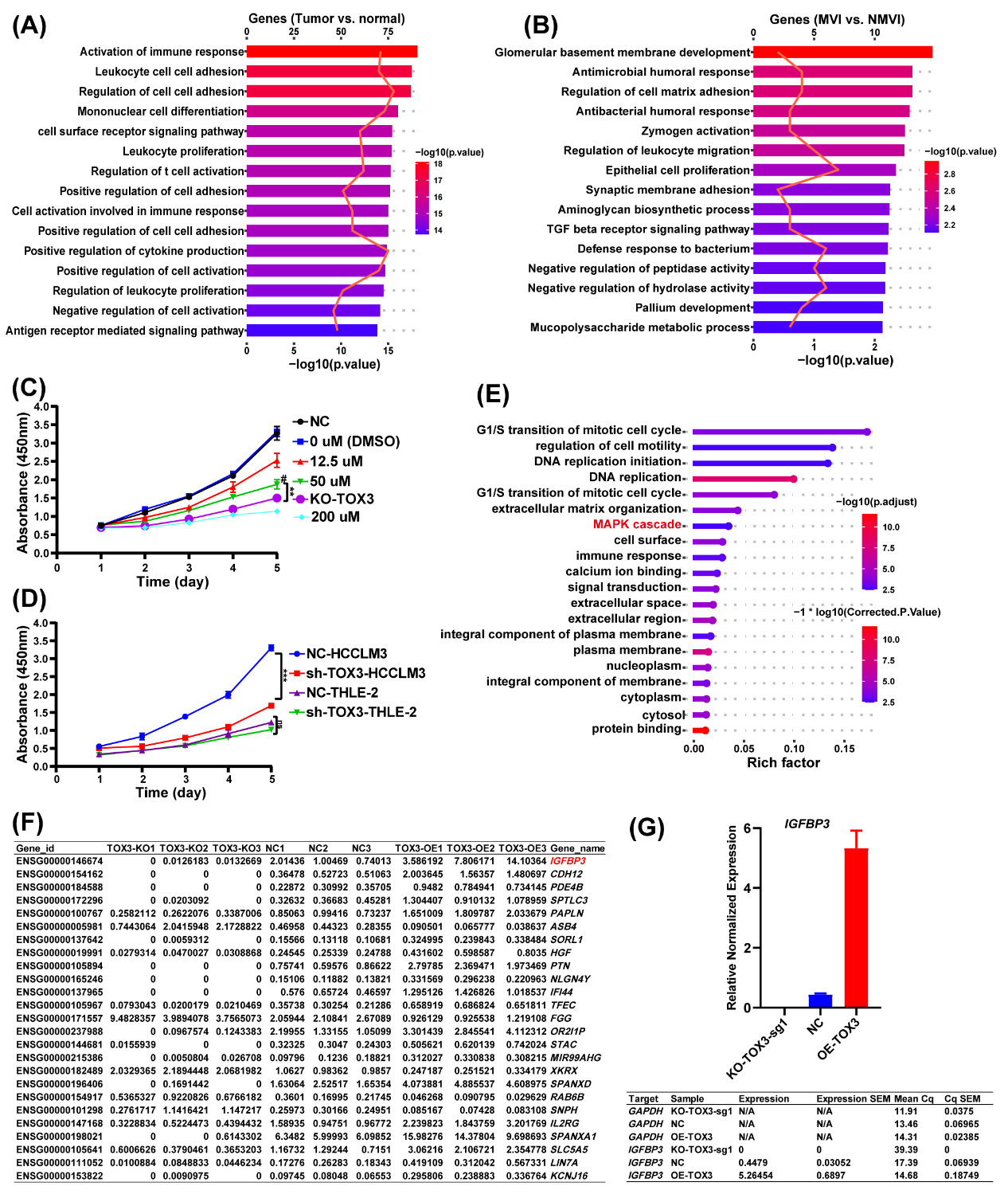
**

**Figure S1: Enrichment analysis of differentially expressed genes and effects of the TOX3 expression level.** (A) and (B) Enrichment analysis (GO_BP gene terms) of the differential genes between HCC and paired normal tissues, and between MVI-positive HCC tissues and MVI-negative HCC tissues, respectively. (C) The inhibitory effect of TOX3 knockout on HCCLM3 cells compared with lenvatinib. ^#^ The IC_50_ of lenvatinib for HCCLM3. (D) Changes in the proliferation of HCCLM3 and THLE-2 cells after TOX3 was knocked down. (E) Enrichment analysis of GO_BP gene terms for genes significantly upregulated in the TOX3-OE group was performed, and the MAPK pathway was found to be significantly enriched. (F) Sequencing revealed that the level of *IGFBP3* was 0 after TOX3 knockout, and the level of *IGFBP3* increased after TOX3 was overexpressed. (G) The sequencing data for *IGFBP3* were confirmed by RT‒qPCR.

**Figure S2**

**
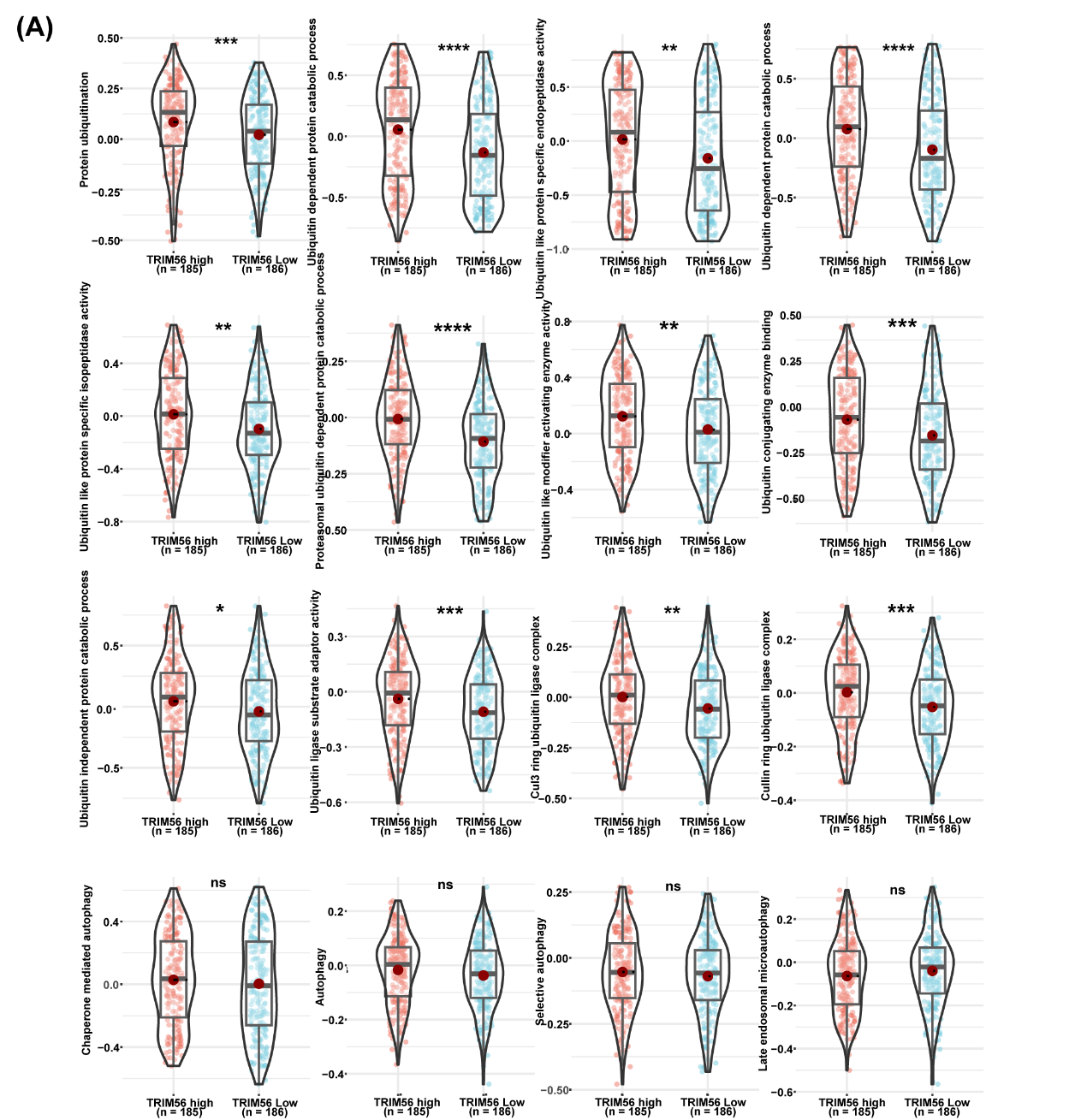
**

**Figure S2: The effects of TRIM56 downregulation.** (A) The public database was used to evaluate the effects of TRIM56 downregulation on the ubiquitin proteasome system and autophagy.

| Variables | Case 1 | Case 2 | Case 3 | Case 4 | Case 5 | Case 6 |
| --- | --- | --- | --- | --- | --- | --- |
| MVI | Negative | Negative | Negative | Positive | Positive | Positive |
| Gender | Female | Female | Male | Male | Male | Male |
| Age (years) | 67 | 38 | 63 | 52 | 50 | 51 |
| BMI, kg/m^2^ | 28.4 | 23.3 | 22.3 | 24.1 | 19.1 | 25.5 |
| HBV carrier | Yes | Yes | Yes | Yes | Yes | Yes |
| HCV carrier | No | No | No | No | No | No |
| AFP，ug/L | 3.85 | 1089.0 | 11.10 | 572.0 | 1210.0 | 1.71 |
| PIVKA-II | 33.0 | 19617.0 | 89.0 | 24968.0 | 3736.0 | 950.0 |
| Tumor diameter (cm) | 2.1 | 5.0 | 2.0 | 15.0 | 5.0 | 12.7 |
| Tumor number | 1 | 1 | 1 | 1 | 1 | 1 |
| Tumor differentiation | Medium | Low | Medium | Low-medium | Low-medium | Medium |
| CNLC stage | Ia | Ia | Ia | Ib | Ia | Ib |
| BCLC stage | A | A | A | A | A | A |
| *TOX3* (normal), count | 120 | 108 | 36 | 119 | 145 | 68 |
| *TOX3* (HCC), count | 145 | 144 | 84 | 721 | 371 | 464 |

**Table S1:** **Patient characteristics of the sequencing group**

Note: HCC hepatocellular carcinoma, MVI microvascular invasion, BMI body mass index, HBV hepatitis B virus, HCV hepatitis C virus, AFP alpha fetoprotein, PIVKA-II protein induced by vitamin K absence or antagonist-II, CNLC China Clinic Liver Cancer staging system, BCLC Barcelona Clinic Liver Cancer staging system.

**Table S2: Relationships between clinical patient data and TOX3 expression in HCC tissues**

| Variables | Total number | TOX3 overexpression | TOX3  low expression | *P* |
| --- | --- | --- | --- | --- |
| Gender |  |  |  | 0.107 |
| Male | 65 | 35 | 30 |  |
| Female | 7 | 1 | 6 |  |
| Age (years) |  |  |  | 1.000 |
| ≥50 | 54 | 27 | 27 |  |
| ＜50 | 18 | 9 | 9 |  |
| Liver cirrhosis |  |  |  | 0.051 |
| with | 45 | 27 | 18 |  |
| without | 27 | 9 | 18 |  |
| Tumor diameter |  |  |  | 0.033 |
| ≥5cm | 36 | 23 | 13 |  |
| ＜5cm | 36 | 13 | 23 |  |
| Tumor number |  |  |  | 0.517 |
| Multiple | 11 | 7 | 4 |  |
| Single | 61 | 29 | 32 |  |
| MVI |  |  |  | 0.009 |
| Positive | 32 | 22 | 10 |  |
| Negative | 40 | 14 | 26 |  |
| Tumor differentiation |  |  |  | 0.018 |
| Low/Low-medium | 35 | 23 | 12 |  |
| Medium/High | 37 | 13 | 24 |  |

Note: MVI microvascular invasion
